# United States Veterinary Medical Association

**Published:** 1890-12

**Authors:** 


### UNITED STATES VETERINARY MEDICAL ASSOCIATION.

A MEETING of the Comitia Minora will be called some time early in January, 1891, to decide upon the place of the next annual meeting. Nominations for the place of meeting can be sent by members to the Secretary, Dr. W. H. Hoskins, 12 S. Thirty-seventh Street, Philadelphia, for consideration by the committee.

### UNITED STATES VETERINARY MEDICAL ASSOCIATION.

RESIDENT STATE SECRETARIES, 1890–1891.

Dr. Ward R. Rowland, Pasadena, California; Dr. Harrison Whitney, Norwalk, Connecticutt; Dr. C. A. Cary, Brooklings, South Dakota; Dr. H. P. Eves, Wilmington, Delaware; Dr. E. S. Walmer, 3229 M St., Georgetown, D. C.; Dr. August Jasme, Atlanta, Georgia; Dr. A. J. Thompson, Evansville, Indiana; Dr. C. E. Hollingsworth, La Salle, Illinois; Dr. S. Stewart, Council Bluffs, Iowa; Dr. D. Lemay, Fort Riley, Kansas; Dr. James L. Kidd, Lexington, Kentucky; Dr. Gerald E. Griffin, Fort Reno, Indian Territory; Dr. F. W. Huntington, Woodford, Maine; Dr. L. H. Howard, Boston, Massachusetts; Dr. E. A. A. Grange, Lansing, Michigan. Dr. R. Price, St. Paul, Minnesota; Dr. M. A. Piche, Fort Custer, Montana; Dr. John S. Meyer, St. Joseph, Missouri; Dr. A. M. Clement, 211 St. Paul St., Baltimore, Maryland; Dr. Wm. H. Lowe, Paterson, N. J.; Dr. W. T. Russell, Nashua, New Hampshire; Dr. Wm. R. Howe, Dayton, Ohio; Dr. S. K. Johnson, 117 W. Twenty-fifth St., New York city; Dr. C. D. McMurdo, Fort Sill, Oklahoma Territory; Dr. W. S. Kooker, 457 N. Fourth St., Philadelphia, Pennsylvania; Dr. James A. McLaughlin, 1 Watermann St., Providence, Rhode Island; Dr. N. McInnes, Charleston, S. C.; Dr. J. W. Schiebler, 312 Second St., Memphis, Tennessee; Dr. I. F. Page, Manchester, Vermont; Dr. John A. Myers, Harrisonburg, Virginia; Dr. V. T. Atkinson, 563 Milwaukee St., Milwaukee, Wisconsin; Dr. Alexander Plummer, Mammoth Hot Springs, Wyoming.

FOREIGN CORRESPONDING SECRETARIES.

Dr. Hara Taha Yokura, Komaba, Tokio, Japan; Dr. J. H. Frinck, St. Johns, New Brunswick; Dr. G. Grimshaw, Kingston, Ontario.

